# A novel algorithm for analyzing drug-drug interactions from MEDLINE literature

**DOI:** 10.1038/srep17357

**Published:** 2015-11-27

**Authors:** Yin Lu, Dan Shen, Maxwell Pietsch, Chetan Nagar, Zayd Fadli, Hong Huang, Yi-Cheng Tu, Feng Cheng

**Affiliations:** 1 Department of Pharmaceutical Science, College of Pharmacy, University of South Florida, Tampa, FL, 33612, USA; 2 Department of Mathematics & Statistics, University of South Florida, Tampa, FL, 33612, USA; 3 Department of Computer Science and Engineering, University of South Florida, Tampa, FL, 33612, USA; 4 College of Medicine, Syrian private university, Damascus, 0100, Syria; 5 School of Information, University of South Florida, Tampa, FL, 33612, USA; 6 Department of Epidemiology and Biostatistics, College of Public Health, University of South Florida, Tampa 33612, USA

## Abstract

Drug–drug interaction (DDI) is becoming a serious clinical safety issue as the use of multiple medications becomes more common. Searching the MEDLINE database for journal articles related to DDI produces over 330,000 results. It is impossible to read and summarize these references manually. As the volume of biomedical reference in the MEDLINE database continues to expand at a rapid pace, automatic identification of DDIs from literature is becoming increasingly important. In this article, we present a random-sampling-based statistical algorithm to identify possible DDIs and the underlying mechanism from the substances field of MEDLINE records. The substances terms are essentially carriers of compound (including protein) information in a MEDLINE record. Four case studies on warfarin, ibuprofen, furosemide and sertraline implied that our method was able to rank possible DDIs with high accuracy (90.0% for warfarin, 83.3% for ibuprofen, 70.0% for furosemide and 100% for sertraline in the top 10% of a list of compounds ranked by p-value). A social network analysis of substance terms was also performed to construct networks between proteins and drug pairs to elucidate how the two drugs could interact.

Drug-drug interaction (DDI) is typically defined as a change in the effect of a drug when it is taken together with another drug. A DDI may involve an increase in the action of either drug, a decrease in drug efficacy, a delay in drug absorption rate, or an unexpected harmful side effect. DDI incidence increases as the simultaneous use of multiple drugs becomes more common^1^,^2^,^3^. Centers for Disease Control (CDC) reported that the percentage of the U.S. population taking three or more prescription drugs increased from 11.8% in 1988–1994 to 20.8% in 2007–2010, and the percentage of people taking five or more drugs increased from 4.0% to 10.1% during this same time period^4^. DDIs are a major cause of morbidity and mortality and lead to increased health care costs. DDIs make up nearly 3% of all hospital admissions^5^ and 3% to 5% of all inpatient medication errors^6^. DDIs are difficult to be observed during clinical trials because clinical trials do not test for DDIs directly. Serious interactions are often discovered after a drug is already on the market for a long time^7^. Therefore, Identification of possible DDIs is very important for drug development and clinical patient care.

The experimental approaches in characterizing DDIs, e.g. *in vitro*^8^, *in vivo*^9^, and *in populo*^10^, are performed at a relatively small scale. Computational methods provide the opportunity to conduct large-scale studies on DDIs. Thus far, computational solutions to predict DDIs have consisted of two distinct approaches: 1) Similarity-based approaches that measure the similarity of drug information. Previous methods were originally designed to infer novel potential targets of drugs based on various type of data, such as molecular structures^11^,^12^, targets^13^,^14^, indications^15^, side-effects^16^ and gene expression profiles^17^. These methods can be used to infer drug interactions. 2) Knowledge-based approaches that predict DDI from scientific literature (biomedical abstracts)^18^,^19^,^20^,^21^,^22^, an electronic medical record database^23^ and the FDA Adverse Event Reporting System^24^, where text mining presents a solution to the problem of uncovering novel DDIs.

DDIs are frequently reported in clinical and scientific journals. Therefore, literature is the most useful and comprehensive resource for the DDI detection. MEDLINE (Medical Literature Analysis and Retrieval System Online) developed by the US National Library of Medicine (NLM), is the most widely used database of life sciences and biomedical literature. MEDLINE contains over 24 million entries from more than 5,600 journals, with 2,000–4,000 new references being added daily. A MEDLINE search for journal articles related to DDI currently produces more than 330,000 results. Though this gigantic amount of MEDLINE literature pertaining to DDI offers an unprecedented opportunity for the study of drug-drug interaction, it is impossible to identifying DDI information from these references manually. High throughput, accurate, reliable and user-friendly retrieval of DDI information is in increasing demand.

Generally, a MEDLINE record representing one journal article is composed of some fields including title of the journal article, author information, journal information, publication type, language, abstract, MeSH terms (Medical Subject Headings), and substances. Recently published research has focused on information extraction from abstract^18^,^19^,^20^,^21^,^22^. In our study we chose the substances field to investigate DDIs because the field of MEDLINE records contains a bounty of useful information of compounds and proteins. These terms in the substances field are essentially carriers of a documents’ information. The Substances terms of MEDLINE records are generally manually indexed by specialized personnel, which makes the information more reliable. To our knowledge, no studies have been performed to investigate DDIs using substances terms from MEDLINE.

Our strategy was to use a random-sampling-based statistical algorithm to compare the co-occurrence of substances with the drug of interest in MEDLINE records containing the keyword “drug interactions” and those in records without “drug interactions”. Four case studies on warfarin, ibuprofen, furosemide and sertraline implied that our method was able to rank possible DDIs with high accuracy. In addition to identifying possible DDIs, the algorithm indicated proteins that may participate in those DDIs. Social network analysis was also performed to construct networks between proteins and drug pairs to elucidate the possible mechanism of DDIs.

## Results

### Identification of the drug pairs with DDIs

For ibuprofen, warfarin, furosemide and sertraline, there are 500, 2013, 650 and 299 articles with DDI-related MeSH terms in the MEDLINE database, respectively. Then, 916, 1364, 932 and 496 candidate substances were identified from the related articles. The cutoff value of compound pair frequency was set at 3, which means that if the frequency of candidate compound was less than 3, the candidate was not considered for further analysis. Our random-sampling-based algorithm showed that the co-occurrences of 39, 161, 62, and 29 chemicals with the corresponding drugs in articles with drug-interactions were significantly higher than in articles without drug-interactions (p < 0.1). As shown in Table 1, in the top 10 compounds identified by our algorithm, 8, 10, 7, 9 compounds were shown to interact with ibuprofen, warfarin, furosemide and sertraline respectively. These four examples show that our algorithm can correctly identify most DDIs.

**Figure 1:**
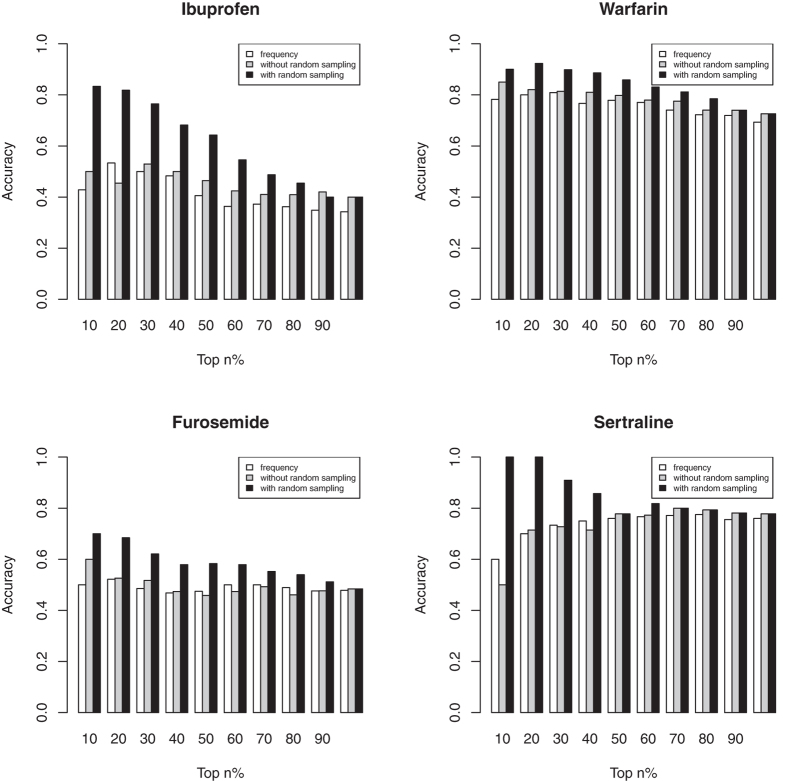
Comparison of performance of three methods (ranking DDIs based on frequency: white, based on co-occurrence without random sampling filtering: grey, and based on co-occurrence with random sampling: black) for ibuprofen, warfarin, furosemide and sertraline.

As shown in Fig. 1, the random sampling step in our algorithm improved accuracy of identification. For example, with such a step, our algorithm ranked DDIs with a high accuracy (90% for warfarin, 83.3% for ibuprofen, 70.0% for furosemide and 100% for sertraline in the top 10% of predicted chemical list, shown in black bars in Fig. 1). However, the accuracy (grey bars) was 85.0% for warfarin, 50.0% for ibuprofen, 60.0% for furosemide and 50.0% for sertraline in the top 10% of compounds identified by co-occurrence without random sampling. The accuracy (white bars) was only 78.3% for warfarin, 42.9% for ibuprofen, 50.0% for furosemide and 60.0% for sertraline in the top 10% of compounds identified by only frequency. The results prove the effectiveness of the random sampling step to filter irrelevant substances terms.

### Analysis of the proteins involved DDIs

Understanding the drug-protein-drug interactions is key in exploring the action mechanism of DDIs. Besides identifying compound pairs that may interact, we also investigated the possible mechanism of DDIs by analyzing the protein information from the substances field of MEDLINE records. Statistically significant proteins from the substances terms were identified using the same random-sampling-based approach. Significant proteins of ibuprofen, warfarin, furosemide and sertraline were shown in Table 2. PTGS1 protein, CYP2C9 and Aryl Hydrocarbon Hydroxylases were identified for ibuprofen. Among them, PTGS1 and CYP2C9 have been shown to be related to DDIs involving ibuprofen. Ibuprofen is a substrate of the hepatic cytochrome isoenzyme CYP2C9^25^. Inhibiting CYP2C9 by other compounds may lead to increased plasma concentrations of ibuprofen^26^. In addition, the adverse gastrointestinal (GI) effects caused by the nonsteroid anti-inflammatory drugs (NSAID) are associated with inhibition of PTGS1^27^. Because ibuprofen exerts similar pharmacologic characteristics to other systemic nonsteroidal antiinflammatory drugs (NSAIDs), additive pharmacodynamic effects, including a potential increase for additive adverse gastrointestinal (GI) effects, may be seen if ibuprofen is used with other NSAIDs^28^.

For warfarin, several cytochrome P-450 (CYP) isoenzymes (including CYP3A4, CYP1A2, CYP2D6 and CYP2C9), steroid 16-alpha-hydroxylase, steroid hydroxylases, aryl hydrocarbon hydroxylases, alanine transaminase, aspartate aminotransferases, PTGS2 and prostaglandin-endoperoxide synthases were identified as statistically significant proteins. Warfarin is stereoselectively metabolized by hepatic cytochrome P-450 (CYP) isoenzymes to inactive, hydroxylated metabolites (predominant route) and by reductases to reduced metabolites (warfarin alcohols). Inhibiting these cytochrome P-450 (CYP) isoenzymes by other compounds may increase or decrease the metabolism of warfarin leading to altered anticoagulation effects^29^. The CYP isoenzymes involved in the metabolism of warfarin include 2C9, 2C19, 2C8, 2C18, 1A2, and 3A4^28^,^30^,^31^,^32^. CYP2C9 is the principle enzyme that metabolizes S-warfarin and modulates the *in vivo* activity of warfarin. The R-isomer is metabolized by CYP1A2 and CYP3A4, and to a lesser extent by CYP2C19.

For furosemide, Thromboxane-A Synthase and Glutathione Transferase were identified. Thromboxane-A synthase inhibition can enhance furosemide-induced renal vasodilation^33^. The activity of Glutathione Transferase might be modified after the interaction between furosemide and sulforaphane, and this may lead to drug effectiveness alternation^34^.

For sertraline, several cytochrome P-450 (CYP) isoenzymes (including CYP2D6, CYP2C9, CYP1A2, CYP2C19), Mixed Function Oxygenases, Steroid 16-alpha-Hydroxylase, Steroid Hydroxylases, Aryl Hydrocarbon Hydroxylases and Receptor, Serotonin, 5-HT1A were identified. *In vitro* data suggest sertraline is a substrate of CYP2B6, CYP2C9, CYP2D6, CYP3A4, and CYP2C19^35^. Inhibiting these cytochrome P-450 (CYP) isoenzymes may increase or decrease the metabolism of sertraline leading to increased serum concentrations and possible toxicity^36^. In addition, antagonizing serotonin (5-HT) receptors could oppose the pharmacologic actions of sertraline^37^. These four case studies show that our algorithm can correctly identify most DDI-related proteins.

### Constructing networks between proteins and drug pairs

**Figure 2:**
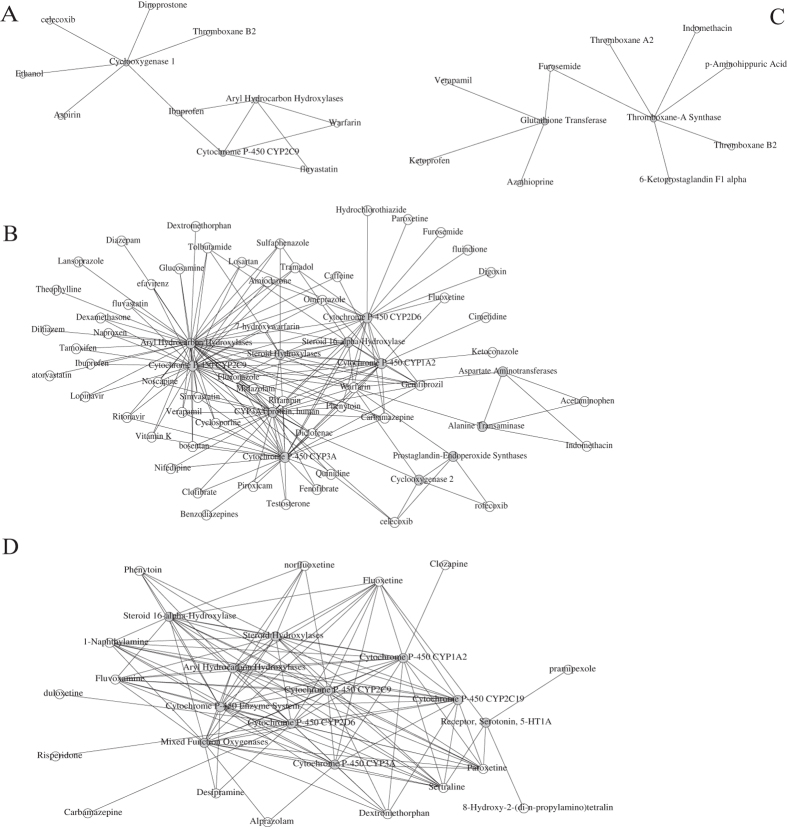
The social network constructed by selected compounds and proteins for ibuprofen (**A**), warfarin (**B**) furosemide (**C**) and sertraline (**D**). The proteins and the compounds are shown in grey and white.

In this study, the relationships between proteins and drugs were modeled using social network analysis. We inferred that the proteins linking drug pairs might take part in the DDIs of the pair. For example, as shown in Fig. 2B, CYP2C9 acted as a link between fluoxetine and warfarin. In clinical studies, combining fluoxetine and warfarin may further increase the risk of bleeding by inhibiting warfarin’s CYP2C9 metabolism^38^. Bosentan interacted with warfarin with CYP2C9 and CYP3A4 as connective nodes. Coadministration of bosentan 500 mg twice daily for 6 days decreases the plasma concentrations of both S-warfarin (a CYP2C9 substrate) and R-warfarin (a CYP3A4 substrate) by 29% and 38%, respectively^39^ The networks will not only show which proteins are more important for DDIs but also provide some mechanistic illuminations. The mechanisms of DDIs may be classified into pharmacokinetic and pharmacodynamic^2^. Pharmacokinetic DDIs occur when one drug influences the ADMET (absorption, distribution, metabolism, or excretion) of another. Pharmacodynamic DDI occur when both drugs act at the same biological target/pathway resulting in synergistic or antagonistic effects. From the constructed network, we find that the DDI-related proteins for warfarin are metabolism-related (such as CYP2C9, CYP3A4, and CYP1A2) and infer that the warfarin involved DDIs are mainly pharmacokinetic.

## Discussion

In this paper we presented a novel algorithm to facilitate knowledge discovery of DDIs by exploring existing high quality information that was manually complied into substances fields in MEDLINE literature. By using a random sampling step our method has the advantage of eliminating irrelevant terms, yielding a high accuracy compared to ranking strategies based on term frequency and term pair co-occurrence without random sampling. In addition, our method appeared to be capable of identifying proteins and networks involved in DDIs, shedding light on potential DDI mechanisms. There are many studies for MEDLINE data mining. Our study innovates in the following ways:

1. Recently published research has focused on information extraction from abstract. We focus on substances terms in MEDLINE databases in this paper. Substance is chosen to investigate DDIs because the field of MEDLINE records contains a lot of useful chemical information including MeSH chemical and drug terms, protocol terms and non-MeSH rare disease terms from the NIH Office of Rare Diseases. The substances terms of MEDLINE records are generally indexed by specialized personnel, which makes the information more reliable. To our knowledge, no studies have been performed to investigate DDIs using substances terms.
2. For four example compounds, the random sampling step in our algorithm yields a higher overall identification accuracy than methods done without random sampling or methods based on frequency only.
3. Our algorithms not only identify possible DDI events but also predict the mechanism by which two (or more) drugs interact. No algorithm is available for identifying the proteins potentially involved in DDIs from the MEDLINE database.

In summary, we believe our algorithm will improve the ability of the research community to efficiently use large and complex MEDLINE data. As the number of records in the MEDLINE database continues to grow, our algorithm will become increasingly vital for DDI studies.

There are some limitations of the proposed algorithm. Information in substances fields is compiled manually, some information is inevitably missing and this is an obstacle for higher recall. Some low-frequency terms were excluded at the cutoff of 3, which may have resulted in lower recall. However, as more and more references are added to MEDLINE, the impact of these factors will be improved in the future. In addition, some mechanism-related information can be summarized in the text of the abstract of articles. Therefore, a text-mining algorithm will be developed in subsequent research to automate extraction of specific information such as, compounds, genes (or proteins) and their associations from the Abstract field of MEDLINE records.

A web service, DDIRANK, will be developed implementing the statistical algorithms described in this paper. Users will query DDIRANK by drug/compound name and view analyzed information from MEDLINE. The service will be freely accessible and support all common Internet browsers. It will be a valuable tool for clinical pharmacists and basic scientists to analyze DDIs in a reliable way.

Generally, the DDIRANK system is an implementation of algorithms that extract substances from MEDLINE records and then probabilistically score and rank these terms. The DDIRANK system will include two components:

1) DDI_extract. The main function of DDI_extract is to extract substances from MEDLINE records. Users can submit analysis queries through the web interface by inputting a compound name. The compound name and its synonym will be used to build a query to search MEDLINE database through the PubMed search engine. All MEDLINE records containing the queried compound will be downloaded. Substances of the retrieved records will then be extracted and stored in a pre-established MySQL database. 2) DDI_substance. The function of DDI_substance is to identify the most reliable DDI-related substance information from the DDI_extract using the random-sampling-based approach shown in this paper. The p-value can rank the extracted substances and filter the terms that are not related to DDIs.

The web service will be developed following a 3-tier design: users can submit analysis queries through the web interface; the queries will be processed using PHP (middle-tier, application server based on Apache) against a MySQL relational database (back-end, database server). The result of each query will be presented to the user in the web browser or through email. The results of any query can be exported to various popular file formats such as CSV and PDF.

## Methods

### Identification of DDI-related compounds or proteins from substances of MEDLINE

**Figure 3:**
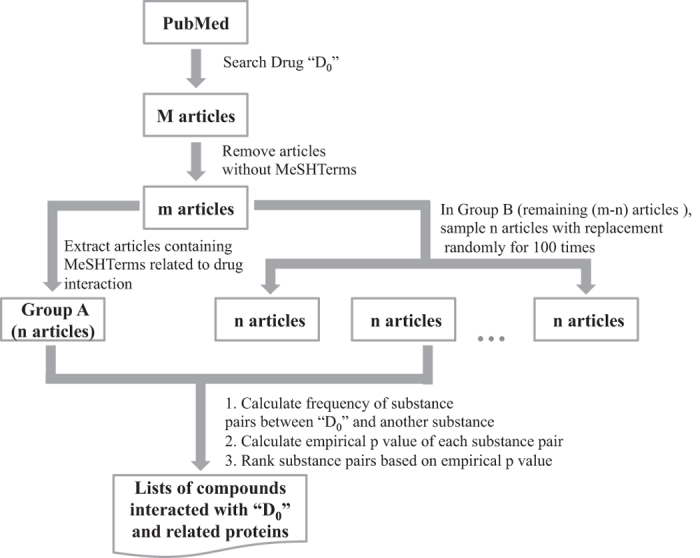
Workflow of the algorithm.

Our proposed algorithm contains the three steps shown in Fig. 3. In the first step, we searched PubMed for the name of the drug and downloaded the resulting articles from MEDLINE. The text in the PubMed IDs (PMIDs), title, abstract, substances and MeSH terms fields of these articles was extracted, excluding those articles that lacked MeSH terms. The remaining articles were separated into two groups: articles containing DDI-related MeSH terms (Group A) and articles without DDI-related MeSH terms (Group B). Here, DDI-related MeSH terms include ‘drug interactions’, ‘drug agonism’, ‘drug partial agonism’, ‘drug antagonism’, ‘drug inverse agonism’, ‘drug synergism’, ‘food-drug interactions’, and ‘herb-drug interactions’. A list of all the compounds and proteins in the substances field of articles in group A was created. From here on we will refer to members of this list as ‘candidates’ and the co-occurrence of a member of this list, either a compound or protein, with the queried drug that was searched for on PubMed, as a ‘term pair’. Then the number of articles that contained a term pair was counted. We set the cutoff value of compound pair frequency at 3, meaning that if the frequency of term pairs in group A was less than 3, the candidate was not considered for further analysis. The value was selected based on the balance of accuracy and the numbers of candidates. As shown in Supplementary Figure 1, with the augment of cutoff, the area under the curve (AUC) increases, while the number of obtained compounds decreases.

Generally, because the articles in group A have DDI-related Mesh terms, we believed the number of compounds and proteins in group A involved in DDIs would naturally be larger than the number of DDI-related compounds and proteins in group B. However, the candidate list derived from the first step contained false positives that were unrelated to DDIs. Therefore, an important task was the exclusion of false positive candidates. In the second step, we compared the number of the term pairs in the substances field of group A with the number of the term pairs in the substances field of group B using random sampling analysis. During this step, the number of articles selected from group B was equal to the number of articles that had been selected from group A. The number of term pairs in group B was calculated. The process was repeated many times (for example, 100 times) to establish the null distribution of the term pairs in the articles without DDIs.

In the third step, the p-value for each candidate compound or protein was calculated based on the distribution identified in step two. Co-occurrences of protein or compound with queried drugs are integers and we made the assumption that the co-occurrences followed a normal distribution. The co-occurrences of a term pair were set as x0 (in group A), and x_1_, x_2_, x_3_…x_100_, (100 times in group B) respectively. Null hypothesis H of testing was that x0 has the same distribution with {x_1_, x_2_, x_3_…x_100_}. The p-value was defined as the probability for a right tail event under the assumption of H. The formula used to calculate the p-value was p = P(x > x_0_). In practice, there occurred a special situation in the distribution {x_1_, x_2_, x_3_…x_100_} of x_1_ = x_2_ = x_3_ = … = x_100_ = 0. In this case, p was set to 0 and the larger x0 was, the higher the corresponding candidate compound or protein was ranked. Statistical significance level was set to 0.1. A p-value less than 0.1 indicated that the number of the term pairs in articles with DDIs (group A) was significantly higher than that in articles without DDIs (group B).

### Social network analysis

Social network analysis was applied to explore the connections among drug pairs and significant protein terms. Social network analysis is a strategy for characterizing networked structures in terms of nodes (compounds or proteins) and edges (interactions) using graph theory^40^. The term pairs whose p-value < 0.1 and corresponding co-occurrence counts ≥ 3 were selected to build a network using the package igraph in R. The terms and articles were considered as people and groups in a social network. The term-article matrix was then taken as the group membership of people.

### Algorithm Validation

We chose four medications, ibuprofen, warfarin, furosemide and sertraline to demonstrate the applicability of our method by validating our DDI predictions against a gold standard. DDI information for these two compounds was collected from LexiComp (http://online.lexi.com), Clinical Pharmacology (http://clinicalpharmacology-ip.com), Drug InteractionChecker (http://www.drugs.com/drug_interactions.php), and Micromedex (http://micromedex.com) databases as a gold standard for validation. Ibuprofen is considered to be among the safest nonsteroidal anti-inflammatory drug with few side effects, however, combining ibuprofen with some medications, such as aspirin increases the risk of certain side effects. Warfarin is an anticoagulant used in the treatment of thrombosis. Warfarin can have dangerous side effects (for example bleeding) from interacting with certain medicines or even with food. Furosemide is a loop diuretic that inhibits sodium and chloride reabsorption. Some of the drug interactions with furosemide can potentially lead to hearing loss, extremely low blood pressure, or low potassium levels. Sertraline is a selective serotonin reuptake inhibitor commonly used to treat anxiety disorders and major depression. Several potential drug interactions with sertraline can cause symptoms of serotonin syndrome, which can be very dangerous.

## Additional Information

**How to cite this article**: Lu, Y. *et al.* A novel algorithm for analyzing drug-drug interactions from MEDLINE literature. *Sci. Rep.* **5**, 17357; doi: 10.1038/srep17357 (2015).

## Supplementary Material

Supplementary Information

## Figures and Tables

**Table 1:** Performance of the identified compounds in a top 10 list of results based on co-occurrence with random sampling.

Compouds interacted with ibuprofen	Frequency	p-value
***Fluconazole***	7	0
Fluvastatin	3	0
***Voriconazole***	3	0
***Digoxin***	6	0
***Lithium***	6	0
***Captopril***	7	0
***Aspirin***	87	0
***Lisinopril***	3	0
***Warfarin***	15	0
Itraconazole	3	2.22E-16
Compouds interacted with warfarin	Frequency	p-value
***Miconazole***	22	0
***Moxifloxacin***	7	0
***Methyl salicylate***	7	0
***Tramadol***	7	0
***Clarithromycin***	7	0
***Terbinafine***	6	0
***Duloxetine***	4	0
***Fenofibrate***	4	0
***Tolterodine***	3	0
***Pitavastatin***	3	0
Compouds interacted with furosemide	Frequency	p-value
***Diflunisal***	3	0
Clenbuterol	3	0
***Probenecid***	21	0
***Indomethacin***	44	0
***Fentanyl***	3	0
***Ceftazidime***	3	0
Cephaloridine	7	3.00E-15
Aprotinin	3	1.07E-13
***Phenylbutazone***	7	1.51E-11
***Warfarin***	8	2.07E-11
Compouds interacted with sertraline	Frequency	p-value
***linezolid***	4	0
***Phenytoin***	4	0
***Ammonium Chloride***	3	0
***Selegiline***	4	0
***Carbamazepine***	9	0
***Methadone***	4	1.76E-13
***pramipexole***	3	3.81E-09
***5-Hydroxytryptophan***	3	6.59E-09
***aripiprazole***	5	2.61E-08
1-Naphthylamine	71	5.44E-07

Compounds predicted correctly are in italic and bold.

**Table 2:** Significant proteins identified from substances of MEDLINE for ibuprofen, warfarin, furosemide and sertraline.

Drug	Extracted enzyme involved in DDIs	Frequency	p value
Ibuprofen	PTGS1 protein, human	5	1.20E-05
	Cytochrome P-450 CYP2C9	3	7.47E-03
	Aryl Hydrocarbon Hydroxylases	3	1.28E-02
Warfarin	CYP3A4 protein, human	13	0
	Cytochrome P-450 CYP3A	17	0
	Prostaglandin-Endoperoxide Synthases	3	2.32E-11
	PTGS2 protein, human	3	2.32E-11
	Cyclooxygenase 2	3	3.38E-08
	Cytochrome P-450 CYP1A2	5	1.77E-04
	Cytochrome P-450 CYP2D6	5	5.10E-04
	CYP3A protein, human	5	5.65E-04
	Steroid 16-alpha-Hydroxylase	11	4.60E-03
	Steroid Hydroxylases	11	7.25E-03
	Aryl Hydrocarbon Hydroxylases	77	8.92E-03
	CYP2C9 protein, human	67	5.81E-02
	Cytochrome P-450 CYP2C9	67	6.08E-02
	Alanine Transaminase	3	8.32E-02
	Aspartate Aminotransferases	3	8.47E-02
Furosemide	Thromboxane-A Synthase	3	0
	Glutathione Transferase	3	1.78E-15
Sertraline	CYP3A protein, human	8	0
	Mixed Function Oxygenases	14	0
	Cytochrome P-450 Enzyme System	18	0
	Cytochrome P-450 CYP3A	8	0
	Steroid 16-alpha-Hydroxylase	5	0
	Steroid Hydroxylases	5	0
	Aryl Hydrocarbon Hydroxylases	8	0
	Cytochrome P-450 CYP2D6	9	0
	Cytochrome P-450 CYP2C9	4	1.76E-13
	CYP2C9 protein, human	4	1.76E-13
	Cytochrome P-450 CYP1A2	3	2.82E-12
	Cytochrome P-450 CYP2C19	3	4.10E-06
	CYP2C19 protein, human	3	4.10E-06
	Receptor, Serotonin, 5-HT1A	3	6.95E-03
