# Supplementary material for: A novel algorithm for analyzing drug-drug interactions from MEDLINE literature
Source: Sci Rep. 2015 Nov 27;5:17357. doi: 10.1038/srep17357 (PMC4661569; doi:10.1038/srep17357)
Supplement: Supplementary Information [file srep17357-s1.pdf]

SUPPLEMENTARY INFORMATION TO

A novel algorithm for analyzing drug-drug interactions from MEDLINE literature

Yin Lu[1], Dan Shen[2], Maxwell Pietsch[3], Chetan Nagar[1], Zayd Fadli[4], Hong Huang[5], Yi Cheng Tu[3], Feng Cheng[1,6,*]

[1]Department of Pharmaceutical Science, College of Pharmacy, University of South Florida, Tampa, FL, 33612, USA

[2]Department of Mathematics & Statistics, University of South Florida, Tampa, FL, 33612, USA

[3]Department of Computer Science and Engineering, University of South Florida, Tampa, FL, 33612, USA

[4]College of Medicine, Syrian private university, Damascus, 0100, Syria

[5]School of Information, University of South Florida, Tampa, FL, 33612, USA

[6]Department of Epidemiology and Biostatistics, College of Public Health, University of South Florida, Tampa 33612, USA

* Correspondence and requests for materials should be addressed to F. C. (email: fcheng1@health.usf.edu)

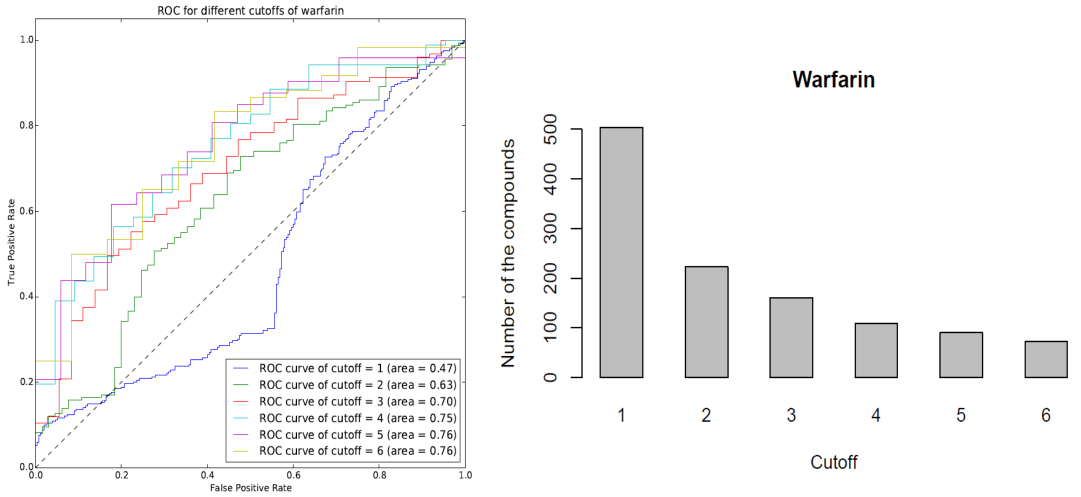

A B

Supplementary Figure S1. ROCs and the numbers of obtained compounds v.s. different values of cutoff when taking warfarin as an example via our algorithm ($p < 0.1$). With the augment of cutoff, the area under the curve (AUC) increases (**A**), while the number of obtained compounds decreases (**B**).
